# Moderate nitrogen application improved salt tolerance by enhancing photosynthesis, antioxidants, and osmotic adjustment in rapeseed (*Brassica napus* L.)

**DOI:** 10.3389/fpls.2023.1196319

**Published:** 2023-05-15

**Authors:** Long Wang, Jingdong Zheng, Guisheng Zhou, Jing Li, Chen Qian, Guobin Lin, Yiyang Li, Qingsong Zuo

**Affiliations:** ^1^ Jiangsu Key Laboratory of Crop Cultivation and Physiology, College of Agriculture, Yangzhou University, Yangzhou, China; ^2^ Joint International Research Laboratory of Agriculture and Agri-Product Safety of the Ministry of Education of China, College of Agriculture, Yangzhou University, Yangzhou, China

**Keywords:** salt stress, rapeseed, nitrogen, photosynthesis, antioxidant, osmotic adjustment

## Abstract

Salt stress is a major adverse environmental factor limiting plant growth. Nitrogen (N) application is an effective strategy to alleviate the negative effects of salt stress on plants. To improve the knowledge of the mechanism of N application on alleviating salt stress on rapeseed seedlings, a pot experiment was conducted with four N application treatments (0, 0.1, 0.2, and 0.3 g N kg^−1^ soil, referred to as N0, N1, N2, and N3, respectively) and exposed to non-salt stress (0 g NaCl kg^−1^ soil, referred to as S0) and salt stress (3 g NaCl kg^−1^ soil, referred to as S1) conditions. The results indicated that in comparison with non-salt stress, salt stress increased the Na content (236.53%) and reactive oxygen species (ROS) production such as hydrogen peroxide (H_2_O_2_) (30.26%), resulting in cell membrane lipid peroxidation characterized by an increased content of malondialdehyde (MDA) (122.32%) and suppressed photosynthetic rate (15.59%), finally leading to inhibited plant growth such as shorter plant height, thinner root neck, lower leaf area, and decreased dry weight. N application improved the plant growth, and the improvement by N application under salt stress was stronger than that under non-salt stress, suggesting that rapeseed seedlings exposed to salt stress are more sensitive to N application and require N to support their growth. Moreover, seedlings exposed to salt stress under N application showed lower ROS accumulation; increased photosynthesis; higher antioxidants such as catalase (CAT), superoxide dismutase (SOD), glutathione reductase (GR), and ascorbic acid (AsA); and greater accumulation of osmotic substances including soluble protein, soluble sugar, and proline, as compared with seedlings without N application. In particular, the best improvement by N application under salt stress occurred at the N2 level, while too high N application could weaken the improvement due to inhibited N metabolism. In summary, this study suggests that moderate N application can improve photosynthesis, antioxidants, and osmoregulation to alleviate the adverse effects of salt stress in rapeseed seedlings.

## 1 Introduction

Rapeseed (*Brassica napus* L.), with a long history of cultivation, is an important oil crop due to its high-quality oil for human consumption. However, salt stress has become a detrimental abiotic stress that limits crop growth and decreases grain yield. It was reported that approximately 7% of the land has been affected by salinity over the world (Munns and Tester, 2008). In particular, approximately 20% of agricultural land and 33% of irrigated agricultural land have been affected by salt, and the total salinized land is increasing at the rate of 10% annually (Shrivastava and Kumar, 2015; Machado and Serralheiro, 2017). Plants exposed to salt stress could suffer from the high concentration of a salt ion in the soil. It was demonstrated that the high concentration of sodium (Na^+^) and chlorine (Cl^−^) in saline soil could destroy the normal physiological process in plants (Zhao et al., 2021). The severity of salt stress relies not only on the concentration of a salt ion but also on the growth stage, such as the seedling stage, flowering stage, or maturity stage. Usually, plant salt tolerance is weaker during early growth stages than in later stages, such as seed germination and seedling growth. Rapeseed plants during the seedling stage are the most sensitive to salt stress during their whole life (Shah et al., 2022). Salt stress occurring during the seedling stage is devastating, resulting in poor growth and development, finally leading to decreasing product because of the weak tolerance of plants against salt stress at the seedling stage. Therefore, more research should be focused on the salt tolerance of rapeseed during a range of growth stages, especially the seedling stage.

Under salt stress, electrons with high energy are transferred to molecular O_2_ to produce the reactive oxygen species (ROS), including superoxide anion (O_2_^−1^), hydroxyl free radicals (OH^−1^), singlet oxygen (^1^O_2_), and hydrogen peroxide (H_2_O_2_) (Rasool et al., 2013; Shahzad et al., 2022). Generally, the production and scavenging of ROS maintain a dynamic balance in the plant (Tuna et al., 2008). However, salt stress could lead to a considerable accumulation of ROS, which exceeds the ability of the elimination system, finally resulting in cell membrane lipid peroxidation. Malondialdehyde (MDA) is the final product of cell membrane lipid peroxidation in the plant. Concurrently, the excessive production of ROS at low concentrations can act as signals to activate the defense system of antioxidants. To counter the damage of ROS accumulation, plants evolved a subtle self-protective system comprised of enzymatic antioxidants (like catalase (CAT), superoxide dismutase (SOD), and glutathione reductase (GR)) and non-enzymatic antioxidants (such as ascorbic acid (AsA)) (Ahmad et al., 2010). In particular, SOD catalyzes the reduction of O_2_^−1^ to H_2_O_2_ and O_2_. H_2_O_2_, which is a toxic substance for the cell, can be reduced to H_2_O by CAT (Dietz et al., 2016). AsA is used as a reductant to reduce H_2_O_2_ to H_2_O (Abogadallah et al., 2011). GR plays an important role in synthesizing glutathione, which is another reductant to reduce ROS (Kaya et al., 2020). Additionally, salt stress could lead to osmotic stress, preventing the plant from absorption of water and nutrients (Chen et al., 2010). In this circumstance, plants can be induced to produce a series of osmotic substances to prevent cell dehydration including soluble protein, soluble sugar, and proline. These osmotic substances play an important role in assisting plants in osmotic adjustment (Hatami et al., 2018).

Photosynthesis is a basal process in plant biomass accumulation and yield formation. Under low salt stress, plants prefer to reduce the stomatal opening to alleviate the damage and maintain normal photosynthesis. However, the ability of self-protection in the plant has certain limitations. Once the salt-ion content in soils reaches a certain level, plant photosynthesis is severely inhibited. Previous studies had been reported that lower water content in plants under salt stress could decrease H_2_O and CO_2_ uptake and therefore reduce photosynthesis (Gomez-Cadenas et al., 2002; Yuan et al., 2014).

Nitrogen (N) is a macro-element for crop growth and production. N is a component of substances including nucleic acids, protein, chlorophyll, and other N metabolites (Zhang et al., 2021). Several N-containing substances can be mediated by salt stress and contribute to the tolerance to salt stress by participating in the eliminating ROS, osmotic adjustment, and recovery of photosynthesis (Dluzniewska et al., 2007; Sudmalis et al., 2018). Plants can absorb two forms of inorganic N sources (ammonium (NH_4_^+^) and nitrate (NO_3_^−^)) from the soil by root system (Dechorgnat et al., 2019). Then, nitrate reductase (NR) and nitrite reductase (NiR) could catalyze NO_3_^−^ into NH_4_^+^, which can be converted into amino acid through glutamine synthetase (GS) and glutamate synthase (GOGAT) (Xu et al., 2012). Nevertheless, salt stress always reduces the N uptake of plants. It was reported that the N accumulation in rapeseed under salt stress was decreased due to the decreased activities of NR, GS, and GOGAT (Wang et al., 2012). Therefore, N application is considered an effective way to enhance the resistance to salt stress and improve plant growth.

Previous studies showed that plant growth was affected by the interaction between N and salt stress, and the effect of N on salt-stressed plants was closely associated with the N rate. For example, excessive N application can effectively alleviate the negative effects of salt stress in maize (Akram, 2014). Similar results were reported in tomatoes that supra-optimum N was more effective to alleviate salt stress as compared with optimum N (Singh et al., 2016). Contrastingly, moderate N application could mitigate the adverse effect of salt stress, while excessive N application aggravated the harmful effect of salt stress in rice and wheat (Ibrahim et al., 2019; Liu et al., 2020). Other studies also showed that excessive N application could cause secondary salinization (Soussi et al., 1998; Lacerda et al., 2016). Therefore, the appropriate rate of N application is the key to alleviating the effects of salt stress, and there was no consistent regular N requirement under salt stress among different species. However, little has been well documented on the interactive effects of salt stress and N on rapeseed plants from the physiological perspective. To this end, the aims of this study were to a) explore the effects of salt stress on the seedling growth and physiological index in rapeseed and b) determine the alleviative effects of N application on rapeseed seedlings through photosynthesis, ROS scavenging system, and osmotic adjustment.

## 2 Materials and methods

### 2.1 Experimental design

This pot experiment was conducted in 2021 and repeated in 2022 at Yangzhou University (32.30°N, 119.43°E), Jiangsu Province, China. The cultivar of Zheyou50, widely grown in China, was used in this experiment. The soils used in this experiment were collected from a plow layer, which was sandy loam with pH 7.1, 1.34% organic matter, 1.2 g kg^−1^ total N, 13.8 mg kg^−1^ available P, and 80.1 mg kg^−1^ available K. Dimension of these plastic pots used in this experiment was 35 cm × 30 cm (height × diameter), and these pots were without holes at the bottom to avoid leakage of nutrient or a salt ion. Each plastic pot contained 10 kg of dry soil.

A completely randomized design was arranged in this study with two levels of soil salinity and four levels of N rate, with 10 pots for each treatment (every pot was considered a replication). The salt stress was achieved by adding sodium chloride (NaCl) into soils, including 3 g NaCl kg^−1^ soil with an equivalent electrical conductivity (EC) of 7.3 dS m^−1^ (referred to as S1), and the soil without added NaCl was considered non-salt stress with an equivalent EC of 0.26 dS m^−1^ (referred to as S0). The relevant quantity of NaCl was dissolved in 3 L of distilled water and was added to the soil in the pots before sowing, and then the saline soil was mixed fully to ensure the saline was well-distributed. The N treatments (as urea fertilizer) included 0, 0.1, 0.2, and 0.3 g N kg^−1^ soil (referred to as N0, N1, N2, and N3, respectively). The N fertilizer was treated as basal fertilizer before sowing. Moreover, all the pots were applied with triple-superphosphate and potassium sulfate fertilizers at the rates of 0.20 and 0.24 g kg^−1^ soil as basal fertilizer before sowing.

Ten seeds with uniform size were sown in each pot on 14 October in every experimental year, which is the usual time when the rape seeds were sown in the field. All the pots were thinned to three plants per pot at the fourth leaf and fifth leaf stages. All the pots were placed in the awning without any screen to keep the temperature, light intensity, and humidity inside the awning consistent with the outside environment. The plastic film above the awning was used to cover the pots when rain was expected and was removed as soon as the rain stopped. Tap water (0.4 dS m^−1^) was used as the source of irrigation during the experiment.

### 2.2 Measurements

#### 2.2.1 Plant growth traits

The seedling was sampled on the 30th day after sowing. The plant height and root neck diameter were measured. The leaf area was determined with a leaf area meter (Model LI-3100, Lincoln, NE, USA). Then, the seedling was dried at 80°C and weighed.

#### 2.2.2 Photosynthesis

Photosynthetic parameters such as net photosynthetic rate (Pn), stomatal conductance (Gs), intercellular CO_2_ concentration (Ci), and transpiration rate (Tr) were measured at the 30th day after sowing using a portable photosynthesis system (LI-6400, USA). The data were obtained from the second and third top leaves from 09:00 to 11:00 on sunny days. The light-saturating photosynthetic photon flux density was set at 1,200 μmol m^−2^ s^−1^. The CO_2_ concentration in the leaf chamber was set at 400 μmol mol^−1^.

The instantaneous carboxylation efficiency (CE) was estimated following the method of da Silva et al. (2019). The formula of CE was as follows:


$$\text{CE}=\frac{\text{Pn}}{\text{Ci}}.$$

#### 2.2.3 Physiological assessment

On the 30th day after sowing, the second and third fully expanded leaves were collected from rapeseed seedlings. Fresh samples were frozen immediately using liquid N and then stored at a low-temperature freezer (−80°C) for physiological measurements, including H_2_O_2_, MDA, SOD, CAT, GR, AsA, soluble protein, soluble sugar, and proline. These physiological traits were determined using commercial enzyme-linked immunosorbent assay (ELISA) kits provided by Shanghai Enzyme-linked Industrial Co., Ltd., Shanghai, China. One gram of fresh leaf sample of rapeseed seedlings was mixed with 9 ml of 50 mM/L phosphate buffer solution (pH = 7.8). Simultaneously, the mixture was added with quartz sand, ground in ice condition, and then centrifuged at 15,000 r/min for 20 min at 4°C. The prepared supernatant and the standard substrate were mixed and reacted for 30 min at 37°C. The plate was washed five times. Then, the enzyme reagent was added and reacted for 30 min at 37°C; after this step, the plate was rinsed five times again. Next, the stain was added for 10 min at 37°C, followed by adding the stop solution. Finally, the optical density (OD) value was read at 450 nm.

#### 2.2.4 N and Na assessment

The leaf N content was determined using the elemental analyzer (Vario MAX CN, Elementar Co., Langenselbold, Germany). The leaf Na content was determined according to the flame photometer method.

#### 2.2.5 Statistical analysis

The results were not statistically different between the two experiments; therefore, the data were pooled for analysis. The analysis of variance (ANOVA) was performed with SPSS Statistics 20 software (SPSS Inc., Chicago, IL, USA). Means were compared by least significant difference (LSD) at the p = 0.05 level. Relationships among rapeseed seedlings’ growth traits, photosynthesis, and physiological parameters were evaluated using Pearson’s correlation analysis. Graphs were prepared using Origin 9.0 software (OriginLab Corp).

## 3 Results

### 3.1 Changes in growth traits and ROS content

**Figure 1:**
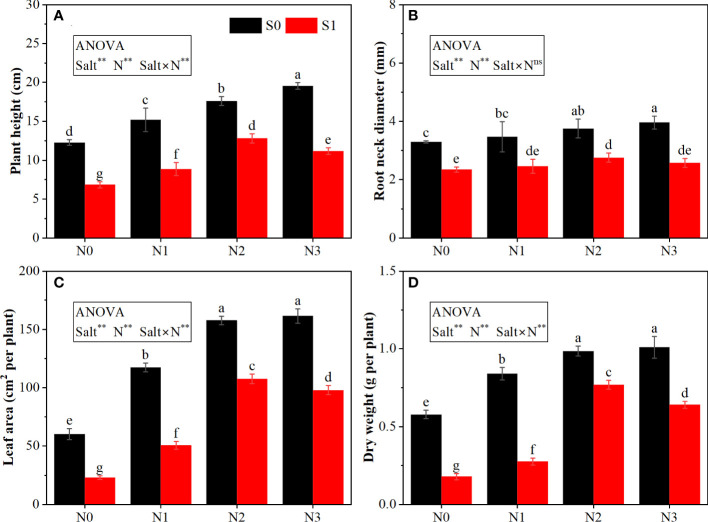
Effects of salt stress on the growth of rapeseed seedlings under different N levels. **(A)** plant height, **(B)** root neck diameter, **(C)** leaf area, and **(D)** dry weight. S0 and S1 respectively represent salinity levels of 0 and 3 g NaCl kg^−1^ soil. N0, N1, N2, and N3 respectively represent N rate levels of 0, 0.1, 0.2, and 0.3 g N kg^−1^ soil. Different letters indicate significant differences at p = 0.05. Probability levels are indicated by ns and ** for not significant and 0.01, respectively.

**Figure 2:**
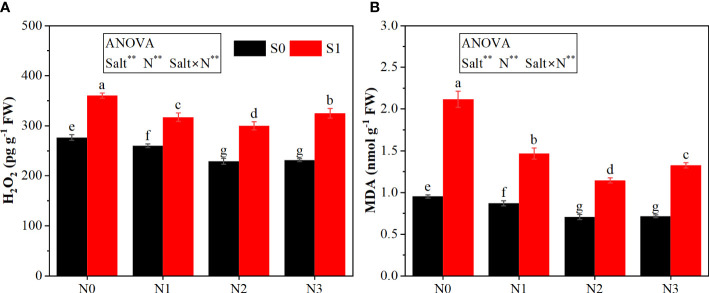
Effects of salt stress on ROS accumulation in rapeseed seedlings under different N levels. **(A)** Hydrogen peroxide (H_2_O_2_) and **(B)** malondialdehyde (MDA). S0 and S1 respectively represent salinity levels of 0 and 3 g NaCl kg^−1^ soil. N0, N1, N2, and N3 respectively represent N rate levels of 0, 0.1, 0.2, and 0.3 g N kg^−1^ soil. Different letters indicate significant differences at p = 0.05. Probability level is indicated by ** for 0.01. FW, fresh weight.

The ANOVA results (**Figures 1**, **2**) indicated that the salt and N significantly affected the plant height, root neck diameter, leaf area, dry weight, H_2_O_2_ content, and MDA content; the interactive effect between salt and N was significant for most parameters except root neck diameter.

Salt stress significantly inhibited the growth of rapeseed seedlings. Under the same N rate, salt stress significantly reduced the plant height, root neck diameter, leaf area, and dry weight of rapeseed seedlings (**Figure 1**) while increasing the content of H_2_O_2_ and MDA (**Figure 2**). As compared with S0N0, the plant height, root neck diameter, leaf area, and dry weight under S1N0 were reduced by 44.30%, 28.64%, 61.99%, and 69.19%, respectively; the contents of H_2_O_2_ and MDA were increased by 30.26% and 122.32%, respectively.

N application improved the seedling growth under both non-salt stress and salt stress. Under the S0 condition, as the N rate increased, plant height, root neck diameter, leaf area, and dry weight increased and peaked at the N3 level, although there was no significant difference between N2 and N3 treatments except plant height. Under the S1 condition, N application enhanced plant growth, which peaked at the N2 level. Moreover, the changes (except root neck diameter) by N application under salt stress were higher than those under non-salt stress. For example, under the S0 condition, the plant height, leaf area, and dry weight under the N2 treatment were 59.35%, 176.08%, and 74.65%, respectively, higher than those under the N0 treatment. Contrastingly, under the S1 condition, the N2 treatment increased these traits by 87.34%, 369.29%, and 331.92% as compared with the N0 treatment. In addition, the N application showed negative effects on H_2_O_2_ and MDA. As the N rate increased from N0 to N2, the content of H_2_O_2_ and MDA under salt stress decreased significantly; then, the contents increased when the N rate continued to increase to N3. Similarly, the alleviative effect of N on MDA content under salt stress was stronger than that under non-salt stress. Under the S0 condition, the N2 treatment reduced the MDA content by 25.76% as compared with the N0 treatment. The reduction under the S1 condition was 45.97%.

### 3.2 Changes in photosynthesis

The ANOVA results (**Table 1**) exhibited that salt, N, and the interaction between salt and N significantly affected Pn, Gs, Ci, Tr, and WUE, except for the interaction on Ci.

**Table 1:** Effects of salt stress on the photosynthesis in the rapeseed seedlings under different N levels.

Salt	N rate (g kg^−1^)	Pn (μmol CO_2_ m^−2^ S^−1^)	Gs (mol H_2_O m^−2^ S^−1^)	Ci (μmol CO_2_ m^−2^)	Tr (mmol H_2_O m^−2^ S^−1^)	CE
S0	N0	11.09 ± 1.02f	0.15 ± 0.01cd	299.17 ± 18.93b	2.77 ± 0.12e	0.037 ± 0.006f
	N1	16.21 ± 0.53d	0.16 ± 0.01c	245.13 ± 15.23d	3.06 ± 0.27d	0.066 ± 0.003d
	N2	23.84 ± 0.79a	0.23 ± 0.01a	221.13 ± 5.33e	3.99 ± 0.21b	0.108 ± 0.006a
	N3	24.22 ± 1.00a	0.24 ± 0.01a	224.05 ± 7.20e	4.31 ± 0.11a	0.108 ± 0.008a
S1	N0	8.85 ± 0.20g	0.11 ± 0.01e	338.03 ± 11.04a	2.05 ± 0.12f	0.026 ± 0.001g
	N1	14.13 ± 1.21e	0.14 ± 0.02d	274.27 ± 10.68c	2.88 ± 0.26de	0.052 ± 0.005e
	N2	21.51 ± 1.17b	0.19 ± 0.01b	250.78 ± 9.36d	3.81 ± 0.17b	0.086 ± 0.008b
	N3	19.49 ± 0.95c	0.18 ± 0.01b	255.20 ± 5.72d	3.52 ± 0.12c	0.076 ± 0.005c
ANOVA						
Salt		**	**	**	**	**
N		**	**	**	**	**
Salt x N		**	**	ns	*	*

Data are mean ± SE (n = 6). S0 and S1 respectively represent salinity levels of 0 and 3 g NaCl kg^−1^ soil. N0, N1, N2, and N3 respectively represent N rate levels of 0, 0.1, 0.2, and 0.3 g N kg^−1^ soil. Different letters within a column indicate significant differences at p = 0.05. Probability levels are indicated by ns, *, and ** for not significant, 0.05, and 0.01, respectively.

Pn, net photosynthetic rate; Gs, stomatal conductance; Ci, intercellular CO_2_ concentration; Tr, transpiration rate; CE, instantaneous carboxylation efficiency.

The salt stress significantly decreased the Pn, Gs, Tr, and CE of rapeseed seedlings. The salt-stressed seedlings showed average reductions of 15.59%, 20.45%, 13.58%, and 25.43% in Pn, Gs, Tr, and CE, respectively, as compared with seedlings under non-salt stress. Conversely, salt stress averagely increased Ci by 13.05%.

N application increased Pn, Gs, Tr, and CE. Under the S0 condition, the Pn, Gs, Tr, and CE significantly increased with the N rate increasing from N0 to N2; there was no significant difference between N2 and N3. Under the S1 condition, the N2 level produced the highest values of Pn, Gs, Tr, and CE, whereas they decreased as the N application increased to N3. Moreover, the increments in Pn, Gs, Tr, and CE by N application under salt stress were greater than those under non-salt stress. For instance, under the S0 condition, the N2 treatment increased Pn, Gs, Tr, and CE by 115.01%, 52.48%, 44.08%, and 189.67%, respectively, as compared with the N0 treatment, while these increments under the S1 condition were 143.07%, 75.20%, 86.24%, and 228.09%, respectively. The Ci showed a constant decrease as the N rate increased from N0 to N2, and there was no significant difference between N2 and N3 under both S0 and S1 conditions.

### 3.3 Changes in antioxidants

**Figure 3:**
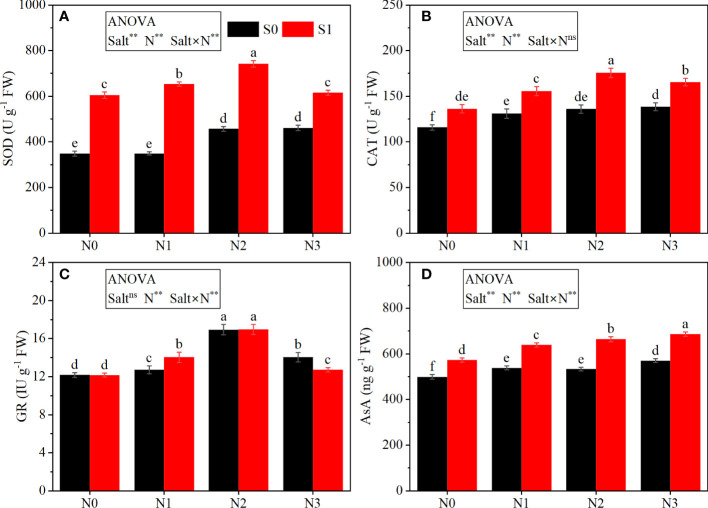
Effects of salt stress on antioxidants in rapeseed seedlings under different N levels. **(A)** Superoxide dismutase (SOD), **(B)** catalase (CAT), **(C)** glutathione reductase (GR), and **(D)** ascorbic acid (AsA). S0 and S1 respectively represent salinity levels of 0 and 3 g NaCl kg^−1^ soil. N0, N1, N2, and N3 respectively represent N rate levels of 0, 0.1, 0.2, and 0.3 g N kg^−1^ soil. Different letters indicate significant differences at p = 0.05. Probability levels are indicated by ns and ** for not significant and 0.01, respectively. FW, fresh weight.

The ANOVA results (**Figure 3**) showed that salt stress and N significantly affected the activities of enzymes (SOD, CAT, and GR) and the AsA content, except salt on GR activity; the interaction between salt and N significantly affected all these parameters, except CAT activity.

Salt stress increased the activities of enzymes (SOD and CAT) and the AsA content. As compared with S0, the S1 treatment averagely increased the activities of SOD and CAT by 64.11% and 21.25%, respectively, and increased the AsA content by 19.62%. The effect of salt stress on GR activity depended on the N rate. Generally, salt stress significantly increased the GR activity under the N1 level, while it significantly decreased under the N3 level. Under the N0 and N2 levels, salt stress had no significant effect on GR activity.

N application increased the activity of antioxidant enzymes and the content of non-enzymatic antioxidants under both non-salt and salt stress conditions. Under the S0 condition, the activities of SOD and CAT, and the AsA content increased with the increase of the N rate and had the highest value in the N3 level. The activity of GR increased as the N rate increased from N0 to N2 and then decreased at N3. However, under the S1 condition, the activities of SOD, CAT, and GR showed a significant increase when N application increased from N0 to N2 and then significantly decreased at the N3 level, except the AsA content, which showed a constant increase with the increase in N application.

### 3.4 Changes in osmotic substances

Salt and N significantly affected the content of soluble protein, soluble sugar, and proline; the interaction between salt and N also had a significant effect on these parameters (**Table 2**).

**Table 2:** Effects of salt stress on the osmotic substances in the rapeseed seedlings under different N levels.

Salt	N rate (g kg^−1^)	Soluble protein (mg g^−1^ FW)	Soluble sugar (mg g^−1^ FW)	Proline (ng g^−1^ FW)
S0	N0	5.37 ± 0.20f	96.33 ± 2.97d	182.26 ± 5.46f
	N1	5.42 ± 0.14f	96.67 ± 2.25d	196.42 ± 8.37e
	N2	7.54 ± 0.12e	104.50 ± 2.69b	232.14 ± 6.71d
	N3	7.80 ± 0.15d	100.57 ± 3.02c	258.48 ± 5.37c
S1	N0	7.82 ± 0.08d	96.64 ± 2.38d	252.28 ± 6.13c
	N1	8.12 ± 0.18c	102.12 ± 3.06bc	270.18 ± 8.19b
	N2	8.80 ± 0.17a	117.22 ± 3.25a	312.99 ± 7.51a
	N3	8.55 ± 0.23b	114.64 ± 2.77a	308.62 ± 6.78a
ANOVA				
salt		**	**	**
N		**	**	**
Salt x N		**	**	**

Data are mean ± SE (n = 6). S0 and S1 respectively represent salinity levels of 0 and 3 g NaCl kg^−1^ soil. N0, N1, N2, and N3 respectively represent N rate levels of 0, 0.1, 0.2, and 0.3 g N kg^−1^ soil. Different letters within a column indicate significant differences at p = 0.05. Probability level is indicated by ** for 0.01.

FW, fresh weight.

Salt stress improved the synthesis of these osmotic substances. As compared with S0, S1 averagely increased the content of soluble protein, soluble sugar, and proline by 30.37%, 8.03%, and 32.55%, respectively. N application further increased the content of soluble protein, soluble sugar, and proline. Under the S0 condition, the content of soluble protein and proline peaked at the N3 level, while the highest soluble sugar content was recorded at the N2 level. Under the S1 condition, the content of soluble protein, soluble sugar, and proline increased significantly as the N rate increased from N0 to N2, and marginal decreases were produced at the N3 level.

### 3.5 Changes in N metabolism

**Figure 4:**
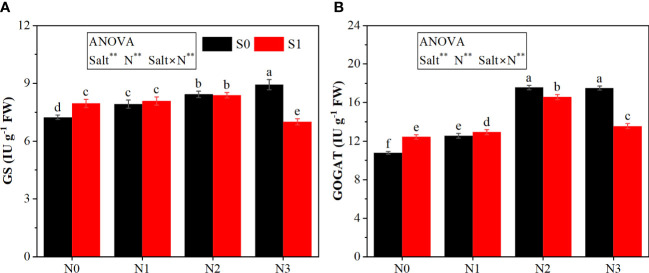
Effects of salt stress on N metabolism in rapeseed seedlings under different N levels. **(A)** Glutamine synthetase (GS) and **(B)** glutamate synthase (GOGAT). S0 and S1 respectively represent salinity levels of 0 and 3 g NaCl kg^−1^ soil. N0, N1, N2, and N3 respectively represent N rate levels of 0, 0.1, 0.2, and 0.3 g N kg^−1^ soil. Different letters indicate significant differences at p = 0.05. Probability level is indicated by ** for 0.01. FW, fresh weight.

The ANOVA results (**Figure 4**) indicated that salt and N and their interaction significantly affected the activities of GS and GOGAT.

The effects of salt stress on N metabolism depended on the N application rate. Under N0 and N1 levels, salt stress increased the activities of GS and GOGAT. However, under N2 and N3 levels, salt stress showed negative effects. In particular, under the N3 level, S1 treatment decreased the activities of GS and GOGAT by 21.49% and 22.57%, respectively.

N application enhanced the activities of GS and GOGAT, with different change tendencies under between S0 and S1 conditions. In particular, under the S0 condition, the GS activity showed a constant increase with the increasing N rate and peaked at the N3 level, and the activity of GOGAT peaked at the N2 level, which was comparable to that at the N3 level. Conversely, under the S1 condition, the activities of GS and GOGAT increased with the increase in N from N0 to N2 and then decreased at N3.

### 3.6 Changes in N and Na contents

The ANOVA results (**Table 3**) indicated that N significantly affected leaf N content, while salt showed no significant effect on it. N, salt, and their interaction significantly affected leaf Na content. The leaf N content increased significantly as the N rate increased from N0 to N2, and there was no difference between the N2 and N3. Regarding leaf Na content, salt stress significantly increased it by 236.53%. However, the N application reduced the leaf Na content under both S0 and S1 conditions. Under the S0 condition, leaf Na content with the N2 treatment was 22.40% lower than the N0 treatment. This reduction was 34.00% under the S1 condition.

**Table 3:** Effects of salt stress on the leaf N and Na contents in the rapeseed seedlings under different N levels.

Salt-ion concentration	N rate (g kg^−1^)	Leaf N content (mg g^−1^ DW)	Leaf Na content (mg g^−1^ DW)
S0	N0	31.98 ± 0.52c	2.18 ± 0.02e
	N1	41.51 ± 0.73b	1.89 ± 0.05f
	N2	45.68 ± 0.61a	1.69 ± 0.03g
	N3	46.22 ± 0.97a	1.66 ± 0.04g
S1	N0	32.01 ± 0.56c	7.33 ± 0.09a
	N1	41.90 ± 0.92b	5.58 ± 0.10b
	N2	45.39 ± 0.84a	4.84 ± 0.08d
	N3	45.46 ± 0.64a	5.07 ± 0.06c
ANOVA			
Salt-ion concentration (SC)		ns	**
N rate (NR)		**	**
Salt x N		ns	**

Data are mean ± SE (n = 6). S0 and S1 respectively represent salinity levels of 0 and 3 g NaCl kg^−1^ soil. N0, N1, N2, and N3 respectively represent N rate levels of 0, 0.1, 0.2, and 0.3 g N kg^−1^ soil. Different letters within a column indicate significant differences at p = 0.05. Probability levels are indicated by ns and ** for not significant and 0.01, respectively.

DW, dry weight.

### 3.7 Correlation analysis

**Figure 5:**
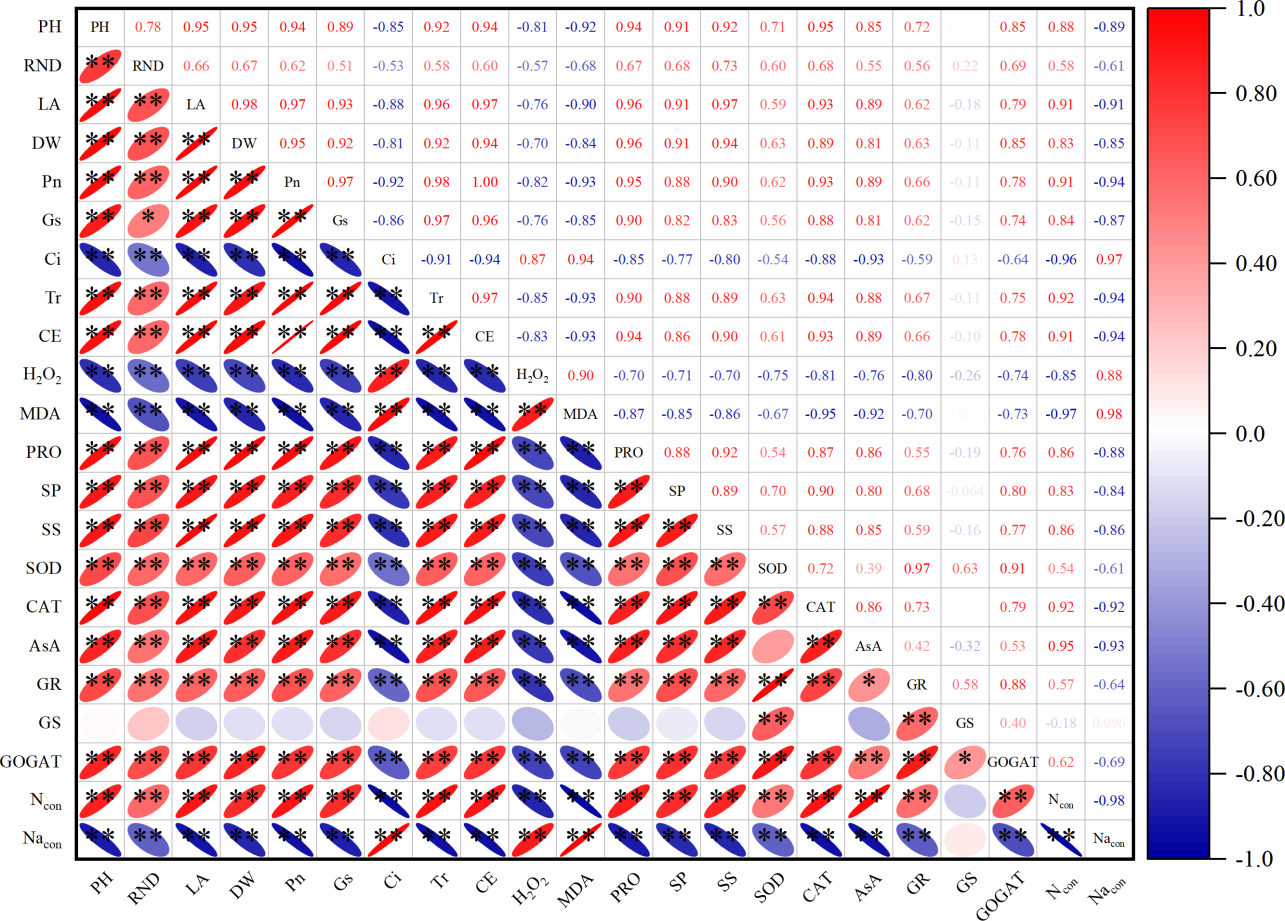
Pearson’s correlation analysis of plant growth, ROS, photosynthesis, antioxidant, osmoregulation, and N metabolism under salt stress. PH, plant height; RND, root neck diameter; LA, leaf area; DW, dry weight; Pn, photosynthetic rate; Gs, stomatal conductance; Ci, intercellular CO_2_ concentration; Tr, transpiration rate; CE, instantaneous carboxylation efficiency; H_2_O_2_, hydrogen peroxide; MDA, malondialdehyde; PRO, proline; SP, soluble protein; SS, soluble sugar; SOD, superoxide dismutase; CAT, catalase; AsA, ascorbic acid; GR, glutathione reductase; GS, glutamine synthetase; GOGAT, glutamate synthase; N_con_, leaf N content; Na_con_, leaf Na content; ROS, reactive oxygen species. Probability levels are indicated by * and ** for 0.05 and 0.01, respectively.

The results of correlation analysis (**Figure 5**) showed that the plant height, root neck diameter, leaf area, dry weight, and photosynthetic rate were negatively related to the content of H_2_O_2_, MDA, and Na under salt stress, whereas they were positively related to the activities of SOD, CAT, and GR and content of AsA, soluble protein, soluble sugar, proline, and N. Moreover, the activities of GOGAT and leaf N content showed a positive relationship with the activities of enzymes (SOD, CAT, and GR), AsA content, photosynthetic rate, and content of soluble protein, soluble sugar, and proline.

## 4 Discussion

At present, salt stress frequently occurs around the world. Although rapeseed is considered a moderate salt-tolerant crop, its physiological and biochemical processes are disrupted under a heavy saline environment (Wang et al., 2022). In this study, we found that salt stress inhibited rapeseed seedling growth and photosynthesis while increasing Na content, ROS accumulation, and product of cell membrane lipid peroxidation as well as improved the antioxidants and accumulation of osmotic substances. N application alleviated the adverse effect of salt stress on photosynthesis, ROS metabolism, and osmoregulation, therefore enhancing seedling growth.

Salt stress always inhibits plant growth symbolized by suppressed leaf expansion and lower biomass accumulation, which could be alleviated by N application (Tian et al., 2022). In our study, the plant height, root neck diameter, leaf area, and dry weight under salt stress were significantly decreased as compared with those under non-salt stress (**Figure 1**). That may be due to altered and disrupted cell division and elongation under salt stress (Pitann et al., 2009). However, N application increased these traits with the highest increments recorded at N2. Moreover, these improvements by N application under salt stress were greater than those under non-salt stress, indicating that seedlings exposed to salt stress are more sensitive to N application and require N to support their growth.

Photosynthesis is a carbon fixation process that is closely related to biomass accumulation and affected by salt stress and N application. In the previous study, salt stress decreased plant H_2_O and CO_2_ uptake by reducing stomatal opening and therefore reducing photosynthetic rate (Sikder et al., 2020). Contrastingly, different results in our study showed that salt stress decreased Gs, whereas it increased Ci, indicating that CO_2_ supply was not decreased because of the effect of salt stress on stomatal and already exceeded that required for photosynthesis. CE, an important indicator of Rubisco activity in the photosynthesis system, was decreased under salt stress. Normally, the reduction of photosynthesis under salt stress could be attributed to the co-effects of stomatal limitation and non-stomatal limitation. These findings demonstrated that non-stomatal limitation might be the main reason for the inhibited photosynthesis in rapeseed seedlings under salt stress, such as photosynthetic enzymatic activity. Similar results were reported that suppression in photosynthesis under salt stress could be correlated with non-stomatal limitations, such as decreases in the activity of RuBP carboxylase and chlorophyll degradation (Xu et al., 2019). Moreover, our results indicated that the decreased Gs and Tr under salt stress might be associated with a tolerance mechanism. Studies had demonstrated that most Na^+^ in plants under salt stress is absorbed by the transpiration stream (Moradi and Ismail, 2007). Therefore, reduced Tr through closing stomatal may inhibit Na^+^ absorption, which can protect plants from salt stress. N is a basic element for the synthesis of protein and plays an important role in plant photosynthesis. Expectedly, N application improved Pn, Gs, Tr, and CE to strengthen the photosynthesis system by mitigating the adverse effect of salt stress on physiological processes such as chlorophyll synthesis, recovery of photosynthetic enzymes, and stomatal opening (Fatma et al., 2016; Lin et al., 2017). We also found that the best enhancement was shown at N2. When the N rate was too high, photosynthesis was inhibited under salt stress. This result may be due to the fact that N metabolism in high N application was suppressed under salt stress (**Figure 4**), resulting in the lower activity of enzymes and lower chlorophyll content in the photosynthesis system. These results demonstrated the importance of the appropriate application of N under salt stress for rapeseed seedlings.

Salt stress has been demonstrated to break the balance of ROS between production and elimination and to result in excessive accumulation of ROS, such as H_2_O_2_ (Rasool et al., 2013). Excessive ROS could lead to cell membrane lipid peroxidation and damage to the membrane integrity. In our study, the content of H_2_O_2_ was increased significantly under salt stress. MDA, an indicator of lipid peroxidation, was also increased markedly. However, the application of N decreased the content of H_2_O_2_ and MDA, with the optimum mitigative effect observed at N2. This may be due to the decreased production of ROS and improved ability of ROS scavenge under N application (Singh et al., 2019). Concurrently, ROS could act as signals to stimulate the self-defense system to eliminate excessive ROS such as SOD, CAT, GR, and AsA. These antioxidants were all increased under salt stress, further promoted by N application, and negatively correlated to the contents of H_2_O_2_ and MDA under salt stress, which indicated that N application could alleviate the oxidative stress under salt stress by enhancing the ability to eliminate ROS. Moreover, in this study, most of these antioxidants were improved with the increase of N rate and peaked at N2, whereas the high N application decreased these antioxidants. It was consistent with the results of Tian et al. (2022), who reported that excessive N application under salt stress could decrease antioxidant activity because of an imbalance of nutrients. Conclusively, moderate N application can effectively enhance the ability of ROS scavenge through improving SOD, CAT, GR, and AsA, whereas excessive N application could weaken the capacity of ROS elimination.

Salt stress could result in osmotic stress, which decreases the ability of the root to absorb water. Plants exposed to salt stress attempt to accumulate more osmolytes such as soluble protein, soluble sugar, and proline to counter the damage of osmotic stress induced by salt stress (Hussain et al., 2021). Therefore, in our study, the content of these osmotic substances was increased, especially soluble protein and proline. N application enhanced the accumulation of these osmotic substances. It was worth noting that under the non-salt stress condition, the soluble protein and proline showed a constant increase with the increasing N rate, whereas soluble sugar was first increased and then decreased. These results may be due to the fact that excessive N application could disrupt the balance of carbon (C) and N metabolism, resulting in more C metabolites being allocated to N metabolism, to provide a C skeleton for the synthesis of protein; therefore, the products of C metabolism decreased. N application enhanced the accumulation of these osmotic substances in salt-stressed seedlings, and the best improvement was recorded at N2, suggesting that moderate N application can help plants to improve the osmoregulation to adjust salt stress, while too high N application under salt stress led to the decrease of osmotic substances through inhibited photosynthesis and N metabolism. Similar results were reported that N supply could increase the accumulation of osmotic substances to enhance the ability of water absorption and salt tolerance (Iqbal et al., 2015; Ali et al., 2021). Generally, the improvement of osmotic substances by N application assists rapeseed seedlings in absorbing water and nutrients to ensure plant growth under salt stress.

Overall, this study found that salt stress increased Na content and ROS accumulation, resulting in lipid peroxidation, and inhibited photosynthesis, finally leading to poor plant growth. N application alleviated the adverse effects of salt stress by improving photosynthesis, antioxidants, and osmotic adjustment. The best improvement by N application under salt stress occurred at the N2 level, while the excessive N application could weaken the salt tolerance because of the decreased N metabolism.

## 5 Conclusion

N application could alleviate the negative effects of salt stress by improving photosynthesis, antioxidants, and osmotic substances, which finally enhanced rapeseed seedling growth. The improvement under salt stress by N application was greater than that under non-salt stress, suggesting that rapeseed seedlings exposed to salt stress are more sensitive to N application and require N to support their growth. In particular, the N application enhanced the activities of enzymatic antioxidants (SOD, CAT, and GR) and the content of non-enzymatic antioxidants (AsA) to eliminate the excessive ROS produced by salt stress. Seedlings with N application under salt stress showed improved osmotic substances (soluble protein, soluble sugar, and proline). It was worth noting that the best improvement by N application under salt stress occurred at the N2 level, while too high N application could inhibit N metabolism and weaken the positive effect. This study not only improved the knowledge of the mechanism of N application in alleviating salt stress on rapeseed seedlings but also provided a theoretical basis for N application in saline soil.

## Data availability statement

The raw data supporting the conclusions of this article will be made available by the authors, without undue reservation.

## Author contributions

LW performed the experiments and wrote the manuscript. LW, JZ, CQ, JL, GL, and YL helped with data collection. GZ and QZ advised on the scientific approach and provided background knowledge. QZ supervised all the experiments and provided funding. All the authors contributed to the article and approved the submitted version.
